# Can mRNA COVID‐19 vaccines induce flares of chronic urticaria?

**DOI:** 10.1111/jocd.15443

**Published:** 2022-10-25

**Authors:** Silada Kanokrungsee, Chananchida Naruenatwanich

**Affiliations:** ^1^ Department of Dermatology, Faculty of Medicine Srinakharinwirot University Bangkok Thailand; ^2^ Department of Dermatology, Faculty of Medicine Siriraj Hospital, Mahidol University Bangkok Thailand

**Keywords:** COVID‐19, urticaria, vaccine

To the Editor, chronic urticaria is a common disease characterized by recurrent pruritic wheals with surrounding erythema for >6 weeks.^1^, ^2^ The etiology is unknown in many patients, leading to a diagnosis of chronic idiopathic urticaria or chronic spontaneous urticaria (CSU). Exacerbating factors include stress, environmental conditions, medications, infections, and vaccination.^3^ Since the emergence of the novel coronavirus disease (COVID‐19) in 2019, vaccines have been needed to prevent the spread of the disease and reduce mortality. Several types of potential vaccines against COVID‐19 have since been developed. A cross‐sectional survey‐based study reported the side effects of mRNA‐ and viral vector‐based COVID‐19 vaccines among German healthcare workers. Of the 599 participants, 474 received the mRNA‐based vaccines and 125 patients received a viral vector‐based vaccine. The most prevalent skin‐related side effect was rash (2.8%), followed by urticaria (0.7%) and angioedema (0.7%).^4^ This study showed that urticaria is one of the most common cutaneous side effects after COVID‐19 vaccination. In 2021, Alflen et al.^3^ reported two cases of well‐controlled CSU triggered by the Moderna COVID‐19 vaccine. However, limited study has confirmed the potential aggravation of mRNA vaccines in patients with chronic urticaria.

We performed a prospective observational study of patients with CSU who received the mRNA COVID‐19 vaccine between September 2021 and February 2022. The patients were recruited from the outpatient clinic at Skin Center, Srinakharinwirot University, Bangkok, Thailand, and online public relations. All the patients were over 18 years of age and diagnosed with CSU, which was well‐controlled. Patients with inducible or physical urticaria or severe disease symptoms (Urticaria Activity Score over 7 days; UAS7 >27) were excluded. The study was conducted through telephone interviews. Clinical outcomes were UAS7 at 1 week before the COVID‐19 vaccination (Week 0) and at the first, second, and third week after vaccination (Weeks 1, 2, and 3). This study was approved by the Human Research Ethics Committee of Srinakharinwirot University (EC/233/2564E), and verbal informed consent was obtained from all participants.

Twenty‐eight patients with CSU who received the mRNA vaccine were included; 9 patients were male (32.14%) and 19 were female (67.86%), with a mean age of 41.78 (SD ± 11.50) years. The median disease duration was 3.5 (2,7) years. Disease control by second or third‐generation antihistamine drugs was achieved in all patients. Half of the patients were regularly taking medication, and the other half were taking it as needed. Seventeen patients (60.71%) received the Pfizer‐BioNTech vaccine, and 11 patients (39.29%) received the Moderna vaccine. Twenty patients (89.29%) were on the third dose of the vaccine, and the majority (92.86%) had side effects from the mRNA vaccine (Table 1). The mean UAS7 at baseline was 3.64 (5.30), a range of 0–20, during the week before vaccination (Table 2). After vaccination, the mean UAS7 score significantly decreased at Weeks 1 and 2 (*p* = 0.013 and 0.007, respectively). Nevertheless, the mean UAS7 at Week 3 compared to the baseline did not show a statistically significant difference (*p* = 0.387).

**TABLE 1:** Demographic characteristics of the participating chronic urticaria patients

Characteristics	Overall (*n* = 28)
Sex, *n* (%)	
Male	9 (32.14)
Female	19 (67.86)
Age (years), mean (SD)	41.78 (11.50)
Onset of urticaria (years), median (IQR)	3.5 (2,7)
Frequency of disease (per month), median (IQR)	2 (1,8)
Disease severity, *n* (%)	
Absence of urticaria (UAS7 = 0)	15 (53.57)
Well‐controlled /Mild (UAS7 = 1–15)	12 (42.86)
Moderate (UAS7 = 16–27)	1 (35.71)
Occurrence of angioedema episodes in the past, *n* (%)	
No	25 (89.29)
Yes	3 (10.71)
Current antihistamine drug use, *n* (%)	
Regularly	14 (50.00)
As need	14 (50.00)
Dose of vaccine, *n* (%)	
Second dose	1 (3.57)
Third dose	25 (89.29)
Fourth dose	2 (7.14)
Side effect of the current vaccine, *n* (%)	
No	2 (7.14)
Yes	26 (92.86)
Fever	11 (42.31)
Pain	14 (53.85)
Diarrhea	1 (3.85)

*Note*: UAS7: 7‐day urticaria activity score.

**TABLE 2:** The mean of Urticaria Activity Score 7 compared between the baseline and Weeks 1, 2 and 3

	UAS7, mean (SD)	*p*‐value
Week 0	3.64 (5.30)	ref
Week 1	2.82 (5.20)	0.013
Week 2	2.75 (4.65)	0.007
Week 3	3.36 (4.49)	0.387

*Note*: *UAS7*: Urticaria Activity Score over 7 days.

Our findings suggest that the mRNA COVID‐19 vaccine did not trigger CSU flare‐ups. A recently published study similarly reported that 92% of 160 CSU patients did not experience a relapse in symptoms after vaccination against COVID‐19.^5^ However, our results may be limited owing to the small sample size, and most of our patients had only mild CSU activity. In conclusion, we have demonstrated that the mRNA COVID‐19 vaccine does not induce flares of mild to moderate severity CSU (UAS7 ≤27). These results support the promotion of COVID‐19 vaccination in CSU patients concerned about the vaccine's side effects.

## AUTHOR CONTRIBUTIONS

SK and CN involved in concepts, design the study, acquired the data, prepared and edited the manuscript, and reviewed the manuscript.

## CONFLICT OF INTEREST

The authors have no conflict of interest to declare.

## ETHICS STATEMENT

The study was approved by the Human Research Ethics Committee of Srinakharinwirot University (SWUEC/233/2564E).

## Data Availability

The data that support the findings of this study are available from the corresponding author upon reasonable request.
